# Role of Thromboelastography and Thromboelastometry in Predicting Risk of Hypercoagulability and Thrombosis in Critically Ill COVID-19 Patients: A Qualitative Systematic Review

**DOI:** 10.5152/TJAR.2021.21118

**Published:** 2022-10-01

**Authors:** Sunaina Tejpal Karna, Pooja Singh, Zainab Ahmad Haq, Gaurav Jain, Alkesh Khurana, Saurabh Saigal, Jai Prakash Sharma, Vaishali Waindeskar

**Affiliations:** 1 Department of Anaesthesiology and Critical Care, AIIMS, Bhopal, India; 2 Department of Anaesthesiology and Critical Care, AIIMS, Rishikesh, India; 3 Department of Pulmonary Medicine, AIIMS, Bhopal, India

**Keywords:** COVID-19, coagulation assays, hypercoagulability, rotational thromboelastometry, thromboelastography, thrombosis

## Abstract

Thromboelastography and rotational thromboelastometry are the viscoelastic point of care devices that use whole blood samples to assess coagulation and fibrinolysis. These devices give information from initiation of the coagulation cascade, activation of clotting factors to fibrin cross-linking, and contribution of fibrinogen and platelet to clot strength and clot lysis. Viscoelastic point of care tests are well established in hypocoaguable states like trauma, cardiac surgery, liver transplantation, and their use in critical care settings with coronavirus disease 2019 (COVID-19) is not so well-known. We performed a systematic review of studies on thromboelastography and rotational thromboelastometry and their modifications to assess their role in critically ill patients with COVID-19. Inclusion criteria were any kind of studies using thromboelastography or rotational thromboelastometry during coronavirus disease critical illness published in English. Ninety-three articles, from December 1, 2019, to August 31, 2020, were identified in the initial search, out of which 12 articles (a total of 380 patients) satisfied the inclusion and exclusion criteria. Thromboelastography and rotational thromboelastometry were observed to detect the hypercoagulable changes and fibrinolysis shutdown associated with COVID-19. Hypercoagulability is associated with an increased risk of venous thrombosis and micro-thrombosis. This review identifies the role of thromboelastography and rotational thromboelastometry in studying the mechanisms contributing to coagulopathy and incidence of thrombosis in COVID-19.

## Main Points

- Thromboelastography (TEG) and rotational thromboelastometry (ROTEM) are able to detect a hypercoagulative state and fibrinolytic shutdown in coronavirus disease 2019 patients with findings of higher clot strength and shorter clot propagation time with a prolonged lysis time.
- A hypercoagulable state may be detected early by TEG/ROTEM with serial measurements being more informative in guiding about the trend of coagulation disturbance.
- Fibrinolysis shutdown as detected by viscoelastic tests may help in the prediction risk of thromboembolism.

## Introduction

The healthcare system all over the world has been challenged by the novel coronavirus severe acute respiratory syndrome coronavirus 2 (SARS-CoV-2). In a series of 184 critically ill patients infected with coronavirus disease 2019 (COVID-19), the rate of venous thromboembolism was observed to be 26% and arterial thrombosis was observed to be 3.7%.^1^ In addition to major venous thromboembolic events, arterial complications including myocardial infarctions have been reported in COVID-19.^2^ Thromboembolic complications may be a direct cause of death in COVID-19. Thrombotic microangiopathy was observed in an autopsy series of patients with COVID-19 in the United States.^3^

With the impact of change in coagulation homeostasis on a patient’s clinical condition, it is imperative to monitor coagulation and fibrinolytic system in patients with COVID-19. Prothrombin time (PT)/International normalized ratio (INR)/activated partial thromboplastin time (aPTT) measure the clotting activity of plasma, ignoring other contribution of platelets and fibrinogen whereas platelet count remains usually normal in COVID-19, with fibrinogen values giving only a quantitative measure, rather than a qualitative assessment.^4,5^

Thromboelastography (TEG) and rotational thromboelastometry (ROTEM) are point of care (POC) viscoelastic hemostatic assays that provide an objective graphical evaluation of kinetics of all the stages of coagulation from initiation and propagation of clot formation till the time it reaches maximum strength and finally dissolves.^6,7^,^8-13^ Rubulotta et al^4^ advised use of POC viscoelastic tests in all patients with COVID-19 with severe pneumonia as activation of coagulation and/or fibrinolysis may occur as part of host response. In addition, they postulated that technology such as the TEG could guide the clinician in understanding this disease by defining specific targets for treatment.

We, therefore, conducted a qualitative systematic review of retrospective and prospective studies in critically ill patients with COVID-19 who underwent viscoelastic POC coagulation tests (TEG/ROTEM) and compared them with conventional coagulation tests (PT/aPTT/platelet count) to assess hypercoagulability.

## Clinical and Research Consequences

### Search Strategy

A comprehensive search strategy was developed following a consensus among the co-authors in collaboration with an external expert. Online databases were queried by pairing the keywords “COVID-19” Or “Coronavirus disease” OR “2019-nCoV” with “Thromboelastography” or “Rotational Thromboelastometry” along with their modifications. Three electronic databases Medline (Pubmed), EMBASE, and Google Scholar were searched from December 1, 2019, to August 31, 2020. Full text of all relevant articles was obtained and evaluated. The references of these articles were then hand searched manually. This systematic review was carried out in accordance with Preferred Reporting Items for Systematic reviews and Meta-Analyses (PRISMA) guidelines. 

### Selection Criteria

Inclusion and exclusion criteria were established for studies to be suitable for inclusion in the systematic review to determine the relevance of data since we wanted to study how the TEG/ROTEM has been used in COVID-19.

Inclusion criteria: (1) We included all publications in critically ill patients suffering from COVID-19 and the use of either TEG or ROTEM along with their modifications. (2) Title and abstract in the English language. (3) Retrospective and prospective observational and case-control studies were included where either TEG or thromboelastometry had been used to study coagulation system in patients with COVID illness.

Exclusion Criteria: (1) Animal studies; (2) Viscoelastic tests other than ROTEM/TEG or their modifications; (3) Correspondence, case report, case series, editorial, erratum/correction regarding TEG/thromboelastometry; (4) Articles not in the English language.

### Data Extraction

Three authors searched the electronic databases and screened all the titles and abstracts of the selected articles independently. Reference list of relevant studies was screened to identify any missing publications. Discrepancies were resolved by discussion and consensus. Full text of potentially eligible articles was then retrieved for further study. The full texts of selected articles were then screened. Quality assessment of observational and cross-sectional studies was done using the National Institute of Health (NIH) “Quality Assessment Tool for Observational Cohort and Cross-Sectional Studies,” and any discrepancy was resolved by consensus opinion of the authors. The relevant information was extracted regarding the role of TEG and ROTEM in detecting changes in the coagulation system in critically ill COVID-19 patients admitted to intensive care unit.

## Results

### Literature Review

The search strategy revealed 93 articles from both manual and electronic databases. After removing duplicates, 79 articles were identified. Subsequent screening of titles and abstracts of these articles resulted in 17 potentially relevant papers describing hypercoagulability and thrombosis with the help of parameters of viscoelastic hemostatic assays. The full texts of these articles were assessed on the basis of inclusion and exclusion criteria regarding eligibility for inclusion in the systematic review.

**Figure 1:**
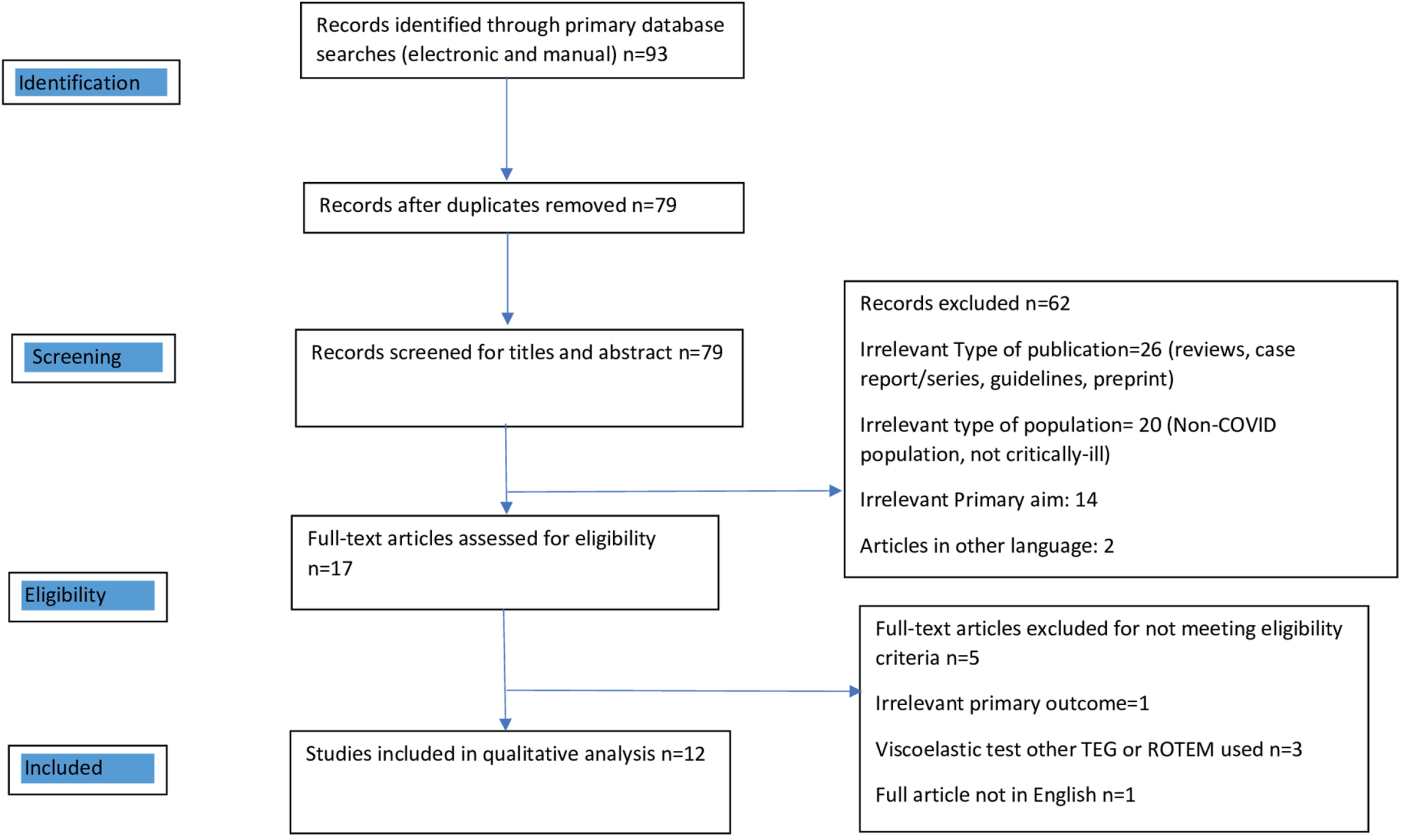
. A PRISMA flow diagram.

A total of 12 articles were included in the quantitative analysis. The details of the selection process and specific reasons for exclusion can be found in the PRISMA diagram (Figure 1). Maximum studies originated from Italy and United States with 4 studies from Italy (1 each from Milan, Florence, Lyon, and Padua), along with 4 from the United States of America (1 each from Houston, Indianapolis, New York, and Denver). One study each was published from Spain, Sweden, London, and Australia. The maximum number of publications, that is, 5 were done in June 2020 followed by 4 in July, 2 in May, and a single publication in August 2020.

### Characteristics of the Studies

A total of 380 patients who participated in 12 studies during the period of February to August 2020 were assessed. Of the 12 included studies, 1 was cross-sectional, 6 were prospective, and 5 were retrospective observational study. All the included studies were single-center studies. 

**Figure 2.**
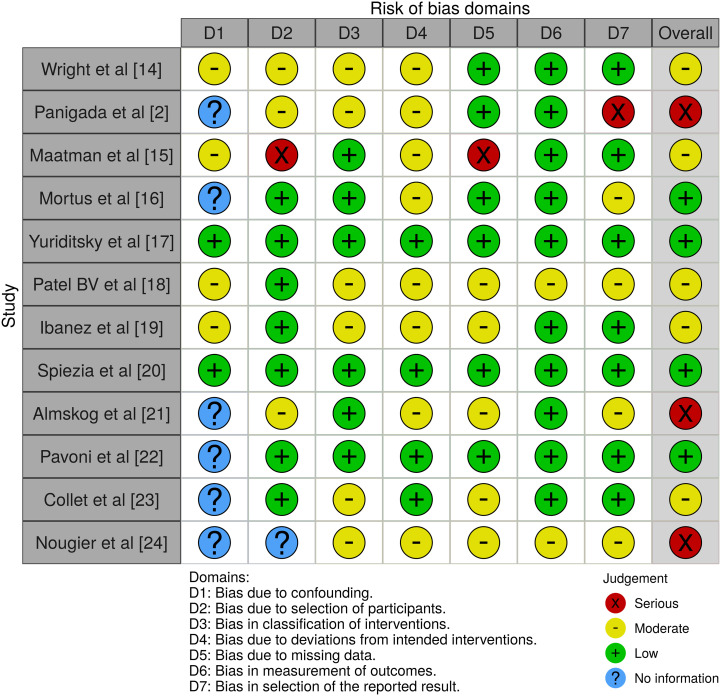
Traffic light plot for assessment of risk of bias.

According to the type of viscoelastic test used in the studies, 6 were based on TEG and 6 on ROTEM. The quality of observational and cross-sectional studies was analyzed using the NIH “Quality Assessment Tool for Observational Cohort and Cross-Sectional Studies” and is presented in Supplementary Table 1. The quality of the studies was mainly fair, but poor in 1 of the studies. The patient populations were homogeneous comprising critically ill patients with positive reverse transcriptase polymerase chain reaction test for COVID-19 infection admitted in COVID intensive care unit (ICU). The follow-up periods for patients were not defined in 2 studies while it was varied in the rest of the studies from 10 days to 2 months. Patient characteristics are presented in Supplementary Tables 2 and 3. Except in 1 study, all the data were obtained from patients admitted in COVID ICU without any comparison group. In 1 study, ROTEM values of patients admitted in COVID ICU were compared with healthy controls. The risk of bias was low in 4, moderate in 5, and serious in 3 trials (Figure 2).^14- 24 [Bibr b17-tjar-50-5-332]^

Modifications of ROTEM used in the studies were as follows: INTEM, evaluation of intrinsic system of coagulation pathway; EXTEM, evaluation of extrinsic coagulation pathways with tissue factor; FIBTEM, evaluation of fibrinogen contribution to blood clot with tissue factor and cytochalasin D which blocks contribution of platelets to maximum clot firmness leaving the impact of fibrin formation and polymerization; and modified TEM-tPA, exogenous tissue plasminogen activator is added to assess hypofibrinolysis.

### ***Definition*** s

Definition of the parameters assessed in the TEG studies was as follows: R time (minutes), which is a measure of the clotting time (CT) from coagulation initiation to the appearance of the clot; K (minutes); α-angle, which is the velocity of clot formation; MA (mm), which is the maximal amplitude of the clot; and Lys-30 is the percentage decrease of clot amplitude at 30 minutes post-MA. Most of the included studies with TEG have defined hypercoagulability as decreased R time, K time, Ly30, and raised α angle as well as MA. Two of the studies have also included coagulation index as a measure of hypercoagulability and values more than 3 were considered significant. 

In ROTEM, the following parameters were analyzed: CT in seconds corresponds to the initiation phase of the clotting process from the beginning of coagulation cascade until the increase in amplitude to 2 mm; clot formation time (CFT) in seconds reflects the measure of the propagation phase of whole blood clot formation with an increase in amplitude starting from 2 to 20 mm; A5 and A10 reflect the clot strength at 5 and 10 minutes; maximum clot firmness (MCF) in mm is the maximum amplitude in millimeters reached in thromboelastogram giving a measure of clot stability; Ly30 is the fibrinolysis at 30 minutes, Ly60 is the fibrinolysis at 60 minutes; and maximum Lysis (ML%) is the measure of fibrinolysis. Most of the included studies in ROTEM have not clearly defined criteria of hypercoagulability. Most studies have suggested that values above or below the reference range will be used to distinguish hypercoagulability. Only one study has defined hypercoagulability as short CFT in EXTEM and high MCF in both EXTEM and FIBTEM. Fibrinolytic shutdown is a phenomenon defined as the presence of elevated D-dimer levels and low fibrinolytic activity. Ly30 < 0-0.8% is a parameter associated with fibrinolytic shutdown and high risk for thrombosis.

### Thromboelastography and Rotational Thromboelastometry, Parameters Associated with Hypercoagulability/Thrombosis

Most of the studies with TEG and ROTEM have assessed the incidence of hypercoagulability in terms of clinical evidence of thrombosis (clinical or CT imaging) while some have assessed hypercoagulability only in terms of numbers of TEG and ROTEM parameters above the reference range. 

Three out of six studies on TEG have calculated the incidence of venous thrombosis ranging from 25% to 43% in patients with hypercoagulable TEG parameters.^14,15,17^ Maatman et al^15^ have estimated that 58% of the patients have at least 1 TEG parameter above the reference range while 83% of the patients have 2 or more TEG parameters above the reference range. Two studies have assessed hypercoagulability in COVID patients directly in terms of hypercoagulable TEG parameters.^2,16,18^ The most common parameters associated with hypercoagulable TEG are high α-angle and MA and decreased Ly30. Mortus et al^16^ concluded that TEG MA has 100% sensitivity and negative predictive value.^16^ Fibrinolytic shutdown denoted by Ly30 <0.8% was found in 5 out of 6 studies^14-18^ (Supplementary Table 2).

Five out of six studies on ROTEM have estimated the incidence of thrombosis, which ranges from 22% to 60% in the patients with hypercoagulable ROTEM parameters.^19,20,22,23^ The most consistent parameter associated with hypercoagulability in EXTEM, INTEM, and FIBTEM assay is decreased CFT and increased maximum clot firmness (MCF). Clot formation time was assessed in 4 out of 6 studies, while MCF was assessed in 5 out of 6 studies showing hypercoagulable values.^2,14,15-18^ Fibrinolytic shutdown denoted by Ly30 = 0 was found in 1 out of 6 studies in EXTEM and FIBTEM assay^14^ (Supplementary Table 3).

***Laboratory Parameters Associated with Hypercoagulability*** D-dimer and fibrinogen levels are consistently increased in all the studies with TEG and ROTEM to assess hypercoagulability in COVID-19 patients. In 2 studies, the D-dimer cut-off of >2600 ng/mL was found to be associated with thrombosis and fibrinolytic shutdown with an area under receiver operator curve of 0.760, sensitivity of 89.7%, and specificity of 59.5%.^14,15,17^ All other laboratory parameters (PT, aPTT, and platelet count) remain within normal reference range and are not associated with an increased risk of thrombosis.

## Discussion

In this systematic review of 12 studies involving 380 patients with severe COVID-19-related critical illness, we observed hypercoagulable TEG/ROTEM findings as evidenced by higher clot strength and shorter clot propagation time in most studies. Another significant finding noted was the presence of fibrinolysis shutdown, which may be a very important contributor toward disease severity. The conventional laboratory tests of PT/INR, aPTT were observed to be within normal limits, with consistent presence of raised D-dimers and fibrinogen in all studies. Though the incidence of venous thrombosis was not analyzed in 3 studies with TEG, it was observed to vary from 25% to 43% in patients with hypercoaguable TEG in the remaining 3 studies,^14,15,17^ with a 36% incidence of microthrombosis reported in 1 study.^14^ Five out of six studies in ROTEM have reported the incidence of thrombosis from 22% to 60%.^19,20,22-24^ However, none of the studies focused on the therapeutic use of TEG/ROTEM to guide treatment. Though we were able to assess our primary outcome of hypercoagulability, it was difficult to conduct a metaanalysis as data collection was at different time points in different studies.

We observed that PT/INR and aPTT, which are frequently used to guide anticoagulant therapy or prognosticate, remained within normal limits in all studies though raised D-dimers and fibrinogen were consistently present. D-dimer indicates in vivo thrombus formation and plasmin-mediated degradation of cross-linked fibrin. Multisystemic manifestations of COVID-19 may be present with elevated circulating D-dimer levels.^25-30^ However, D-dimers test is non-specific and may be affected by age, gender, race, immobilization, hemodialysis, active malignancy, rheumatoid arthritis, sickle cell disease, and prior thromboembolic disease.^31^ In COVID-19 illness, especially in severe patients, the mechanisms of elevated D-dimer or thrombosis may include older age, chronic diseases, hypoxemia, hypercytokinemia, coagulopathy, and inevitable prolonged bed rest.^32^ In a recent meta-analysis, elevated D-dimer and fibrin degradation products (FDP), prolonged PT, and decreased antithrombin predict higher risk stratification and poorer prognosis in COVID-19.^32^ This ambiguity of conventional laboratory tests further sheds light on the significance of POC viscoelastic tests to study characteristics of coagulopathy.

Viscoelastic POC coagulation tests like TEG and ROTEM assess clot formation, strength, and dissolution by measuring the amount of continuously applied rotational force that is transmitted to an electromechanical transduction system and displays the result as a graph. The greatest advantage of POC Venous embolism (VE) assays is the assessment of qualitative function of multiple components of clot formation and lysis in a single rapid assay including platelets, fibrinogen, soluble coagulation factors, and other blood cellular components. Even in non-COVID era, POC viscoelastic assays like TEG and ROTEM have been used in critically ill patients.^33-37^ Endothelial damage was observed to be intimately linked with coagulopathy in severe sepsis as evidenced by an association between higher circulating biomarkers of sepsis and hypocoagulability.^37^ Johansson et al^38^ observed that hypocoagulability as evaluated by TEG was frequent at admission in general ICU patients and associated with a higher rate of ventilator treatment, higher rate of renal replacement therapy, and higher use of blood products. They found that hypocoagulability is an independent risk factor for 30-day mortality. Both procoagulant and anticoagulant states in disseminated intravascular coagulopathy (DIC), as indicated with thrombelastography, were demonstrated to have good correlations with clinically important organ dysfunction and survival. Compared with conventional coagulation tests, TEG/ROTEM can detect impaired fibrinolysis, which can possibly help to discriminate between sepsis and systemic inflammatory response syndrome.^36^

### Mechanism of Coagulation Disturbance in Coronavirus Disease 2019

The coagulation disorder in COVID-19 is complicated. Various theories of increased macro- and microthrombotic complications in critically ill patients with COVID proposed include immune dysregulation, upregulation of ACE2 receptors with direct viral invasion, and perivascular inflammation causing endothelial injury and impairment of fibrinolytic system leading to a prothrombotic state.^39-41^ The coagulation pattern in severe COVID illness appears to be unique as evidenced by the presence of multiple fibrin thrombi within distended small vessels and capillaries and extensive extracellular fibrin thrombi in recent autopsy studies.^42^

In contrast to the hypocoagulable state noted in sepsis in non-COVID patients, hypercoagulability was consistently noted in most studies using TEG and ROTEM in critically ill patients with COVID-19. The mechanism highlighted using viscoelastic tests include findings consistent with faster clot formation (CFT, α angle) and increased clot strength (MCF, MA) along with the presence of fibrinolysis shutdown. So, the hypercoagulability is not just due to hyperfibrinogenemia but also due to impaired fibrinolysis. With the interplay between inflammation, cytokine storm, and subsequent activation of coagulation, SARS CoV-2 is likely to promote massive fibrin formation. Deposition of fibrin in alveolar and interstitial lung spaces along with microvascular thrombosis may worsen respiratory failure. Tissue plasminogen activators (tPAs) have a short half-life (3 minutes), whereas plasminogen activator inhibitor-1 levels are greatly increased.^23^ This was the reasoning proposed by Collet et al^23^ for the unlikely presence of fibrinolysis in COVID-19 patients with cytokine storm. Fibrin deposits are found in lungs due to dysregulation of coagulation and fibrinolytic systems. This approach has been used in few case series where thrombolytics with tPA were used in refractory cases of hypoxia, demonstrating improvement in PaO_2_/FiO_2_ ratio i.e partial pressure of oxygen to fractional inspired oxygen concentration ratio (P/F) ratios with single dose or prolonged infusions.^43-44^ Viscoelastic POC tests may help in monitoring the fibrinolytic activity. 

### Course of Coagulation Disturbance in Coronavirus Disease 2019

It is very important to identify the time of affliction of hypercoagulable state in COVID-19 to guide precise therapy. Viscoelastic tests suggest that coagulation disturbance may be remarkable in the early course of the disease. Pavoni et al studied the temporal association of ROTEM on the day of admission, fifth, and tenth day. They found that though the propagation of clot formation (CFT on INTEM) was more rapid in the first 5 days at admission, it normalized by the 10th day. However, the MCF remained high on INTEM, EXTEM, and FIBTEM even till the fifth and tenth day after admission with a significant decrease in ML% from admission to the tenth day, signifying a decrease in fibrinolysis. Severe COVID pneumonia was observed to be associated with hypercoagulation that persisted over time.^22^ These findings were supported by Almskog et al^21^ who observed that coagulopathy is present early in the course of the disease. Atypical respiratory symptoms observed in COVID-19 may partly be caused by thromboembolism impairing lung perfusion. Rotational thromboelastometry variables may be affected earlier in disease course compared to D-dimers and may be of greater value as predictor of disease severity. In fact, patients with more severe disease may have a more pronounced coagulopathy in COVID-19 patients.^21^

### Prognostication

Thromboelastography may be used in prognostication and prediction of thrombotic episodes. Mortus et al^16^ observed that TEG MA provided 100% sensitivity along with 100% negative predictive value for thrombotic events, although there was no difference in prothrombin time, INR, aPTT, or platelets. Higher thrombotic rates were detected with TEG results outside of the normal reference range, with a 62% thrombosis rate. They also postulated that underdiagnosis or undertreatment of hypercoagulation may explain the high incidence of unexplained COVID-19 mortalities. Wright et al^14^ observed that 40% rate of venous thromboembolism (VTE) was reported in patients with fibrinolysis shutdown compared to 5% in those without. Further, α angle and D-dimer were significantly associated with new-onset need for dialysis. A D-dimer level above 2600 ng mL-1 was associated with higher need for new-onset dialysis (73% vs 17%). They found that a combination of 3 parameters of Ly30 of 0%, D-dimer >2600 ng mL-1, and need for hemodialysis was associated with VTE in 50% and need of hemodialysis in 80% of patients and may serve as a sensitive marker for patients most at risk of VTE and other thrombotic complications.^14^ Similarly, Maatman et al^15^ also suggested that serial use of TEG may provide useful insights to guide which patients are at higher risks for microvascular and macrovascular thrombosis. 

### Therapeutic Guide

None of the included studies used TEG/ROTEM to guide therapy. However, Almskog et al^21^ suggested that if an individual at risk of developing COVID-19-related thrombosis is identified at an earlier stage, enhanced prophylaxis with low molecular weight heparin (LMWH) may decrease mortality in this group of patients. Viscoelastic tests like TEG or platelet function analyzer have also been recommended by the Chinese expert consensus on diagnosis and treatment of coagulation dysfunction in COVID-19. In the theory of the immune-thrombosis relationship where inflammation and thrombin formation are directly correlated, heparin could decrease the inflammatory response by blocking thrombin formation. The American Society of Haematology currently recommends standard prophylactic dose anticoagulation for all COVID-19 patients, reserving therapeutic anticoagulation for those with documented VTE.^45^ Viscoelastic tests of coagulation are easy single bedside tests that can evaluate different components and stages of coagulation and platelet function. Rubulotta et al^4^ have suggested that bedside technologies like TEG can guide clinician in exploring, learning about this new disease, and treating these patients to a more specific target.

The quality of the studies included in the systematic review is suboptimal. Among the 12 studies included, 11 were of fair quality whereas 1 was of poor quality as analyzed using the NIH “Quality Assessment Tool for Observational Cohort and Cross-Sectional Studies.” The different modifications of ROTEM like EXTEM, INTEM, FIBTEM, or TEM tPA are used in different studies. Most of the studies have only documented TEG at a single time point. However, this may not be the optimal measure of a patient’s coagulation profile over the duration of their ICU stay or hospitalization. Most studies have not mentioned the time gap between heparin and POC testing, which may have had an impact on the test result. Similarly, outcomes reported in different studies are different. Though some studies have reported the overall rates of thromboembolic events, true occurrence rates of pulmonary embolism are not known or underestimated as many patients have not undergone computed tomography scan due to logistic issues. 

## Conclusion

This systematic review strongly establishes that TEG/ROTEM helps in the detection of hypercoagulation and fibrinolysis shutdown. Serial TEG/ROTEM over the course of illness may be more informative in guiding about the trend of coagulation disturbance. They can help in understanding the mechanism, time course, and prognostic value of coagulation disturbance in critically ill patients with COVID-19. It is prudent to understand that no test can work in isolation, and any tool is only as useful as the acumen of the clinician for its utilization, for prevention, diagnosis, or to guide therapy. Hence, constant vigilance is recommended. Further randomized controlled trials with more specific standardization of time of testing, repeated evaluation, and response to therapy so as in order to use these tests as a guide to therapy are the need of the hour. The relationship of TEG/ROTEM to thromboembolic events may be more instructive.

## Figures and Tables

**Supplementary Table 1.** Summary of Articles: Quality of the Studies Was Assessed with Quality Assessment Tool for Observational Cohort and Cross-Sectional Studies

S. No.	Authors	Study design	Duration of the Study	Inclusion Criteria	Exclusion Criteria	Type of Viscoelastic Test Used	Modifications of Viscoelastic Test Used	Primary Outcome of Studies	Quality Assessment Grade for Observational Studies
1.	Wright et al (August 2020)	Observational study	March 1, 2020, to April 20^,^ 2020	Patients admitted to COVID ICU	Not defined	TEG	-	Venous thromboembolism and new-onset renal failure prediction	Fair
2	Panigada et al (July 2020)	Observational cohort study	Not defined	Patients admitted to COVID ICU	Not defined	TEG	-	Hypercoagulability with severe inflammatory state	Poor
3.	Maatman et al (May 2020)	Prospective observational study	March 12 to 31, 2020	Critically ill COVID 19 patients admitted in ICU	Patients less than 18 years Imprisoned patients Pregnant patients Patients elected for comfort care	TEG	-	Hypercoagulable in 50%	Fair
4.	Mortus et al (June 2020)	Retrospective cohort study	March 15 to April 19, 2020	Patients admitted to institutional ICU for RT-PCR confirmed COVID 19 infection	Not defined	TEG	With heparinase Without heparinase	Hypercoagulable	Fair
5.	Yuriditsky et al (June 2020)	Retrospective study	April 1 to 20, 2020	Patients admitted to COVID ICU with confirmed RT-PCR test	1.Known hypercoagulable state (i.e., Factor V Leiden) 2. Active malignancy 3. Blood product transfusion within 24 hours of thromboelastography 4.Known history of VTE prior to admissions 5.Thrombocytopenia with platelets less than 150 6.Acute liver failure or history of cirrhosis 7. Currently receiving antiplatelet therapies	TEG	With heparinase Without heparinase	Hypercoagulable	Fair
6.	Patel et al (June 2020)	Retrospective observational study (39)	March 17 to April 10, 2020	Laboratory-confirmed positive COVID-19 infection Mechanically ventilated or on ECMO with active COVID-19 respiratory failure Had undergone CT pulmonary angiography	Not defined	TEG	CK assay	Kaolin heparinase assay (CKH)-MA raised and Ly 30 of 0%	Fair
7.	Ibanez et al (July 2020)	Prospective cohort study (19)	April 5 to 16, 2020	Adult patients admitted to institutional ICU for confirmed COVID-19 infection	Patients on anticoagulant treatment.	ROTEM	EXTEM INTEM	Clot firmness (MCF) raised with decreased fibrinolysis	Fair
8.	Spiezia et al (June 2020)	Prospective observational study (22)	March 7 to 19, 2020	Consecutive patients admitted to institutional ICU for acute respiratory distress syndrome from COVID-19 infection	1.Preexisting congenital bleeding or thrombotic disorders 2. Pre-existing acquired coagulopathies 3. Active cancer and/or chemotherapy 4. Pregnancy 5.Anticoagulant therapy	ROTEM	INTEM EXTEM FIBTEM	Hypercoagulable, shorter CFT in EXTEM and INTEM and higher MCF in all 3	Fair
9.	Almskog et al (June 2020)	Prospective Observational Study (60 cases and 89 controls)	March 8 to 31, 2020	Patients over 18 years of age Patients tested positive for COVID-19 infection and in need of hospital care	Not defined	ROTEM	EXTEM, FIBTEM	EXTEM/FIBTEM-MCF significantly higher than controls and less severely ill EXTEM-CFT shorter than in controls	Fair
10.	Pavoni et al (May 2020)	Retrospective observational study (40)	February 28 to April 10, 2020	Patients ≥ 18 yearsSevere disease with COVID-19 requiring ICU admission	Not defined	ROTEM	INTEM EXTEM FIBTEM	Hypercoagulability	Fair
11.	Collet et al (July 2020)	Retrospective observational point prevalence cohort study (6)	April 2020	Patients ≥ 18 years old admitted to ICUCOVID-19-associated respiratory failure	Not defined	ROTEM	-	Supranormal clot firmness, minimal fibrinolysis	Fair
12.	Nougier et al (July 2020)	Prospective observational study (78) (TEM-tPA used in 23 cases)	Not defined	Adult patients Patients with positive COVID RT-PCR test	Not defined	ROTEM	tPA-modified ROTEM (tPA 0.625 µg ml−1)	Higher MCF and Ly30	Fair

CT, computed tomography; TEG, thromboelastography; ECMO, extracorporeal membrane oxygenation; CFT, clot formation time; COVID, coronavirus disease; ICU, intensive care unit; RT-PCR, reverse transcriptase polymerase chain reaction; MCF, maximum clot firmness; TEM-tPA, modified ROTEM assay by adding exogenous tPA; Ly30, lysis at 30 min; EXTEM, extrinsic coagulation pathway; INTEM, intrinsic coagulation pathway, FIBTEM, fibrinogen function; ROTEM. rotational thromboelastometry.

**Supplementary Table 2.** Studies Showing Hypercoagulability Through Thromboelastography and Laboratory Coagulation Parameters

S. No.	Author	Thromboelastography Parameters					Laboratory Coagulation Parameters			Incidence of Thrombosis and/or Hypercoagulability Coagulation Parameters (TEG and LCP) Associated with Hypercoagulability
R (min)	K (min)	α (˚)	MA (mm)	Ly30 (%)	D-Dimer (ng mL-1)	Fibrinogen (mg dL-1)	Platelet Count (10^9^ L-1)
1.	Wright et al* (n = 44)	5.8 (4.8-8.6)	-	71 (66-74)	73 (67-77)	0 (0-0.4)	1840 (935-8045)	658 (560-779)	232 (186-298)	VTE (25%) Renal failure (microthrombosis) (36%) Ly30 = 0 (*P* = .022) and D-dimer >2600 ng/mL (*P* = .005) is associated with VTE = 50% Hemodialysis = 80%
2.	Panigada et alᶧ (n = 30)	6.3	1.5	69.4	79.1	7.8	4877	680	348	Incidence of thrombosis not analyzed K and Ly30 decreased (90%-100% of cases) α and MA increased (77%-87% of cases) D-dimer and fibrinogen increased (93%-100% of cases)
3.	Maatman et alᶤ* (n=12)	4.8 ± 1.1	1.4 ± 1.1	69.6 ± 10.9	70.8 ± 8.5	0.8 ± 0.9	4046 (3071-13 324)	651 (465-771)	294 (223-340)	Thrombosis in patients with hypercoagulable TEG = 43% Hypercoagulability according to One parameter = 58%Two parameters = 83%
4.	Mortus et al¶ (n = 21)	10 (11)	-	60 (23)	67 (17)	0.9 (1.8)	8300 (7000)	740 (240)	210 (100)	Incidence of thrombosis not analyzed. 74% (14/21) has increased α and MA 28% (5/21) has increased MA only TEG-MA has 100% sensitivity and negative predictive value
5.	Yuriditsky et al (n = 64)	5.25 (4.5-7.6)	0.8 (0.8-1)	77.3 (75.4-79)	76.2 (72.1-81)	0 (0-0.38)	2779 (1972-5575)	711 (496-853)	266 (197-326)	VTE = 31% 50% patients had CI > 3. Decreased R and K times in 43.8% Increased α angle and MA in 70.3% and 60.1%, respectively
6.	Patel et alᶤ (n = 39)	11.3 (4.3-8.3)	1.3 (0.8-1.9)	75.7 (64-77)	70.6 (52-69) (CKH-MA) 48.5 (15-32) (Citrated functional fibrinogen [CFF]-MA)	0 (0-2.2)	6440 ± 10 434	660 ± 190	272 ± 77	Incidence of thrombosis not analyzed Raised MA and absence of fibrinolysis. 54% (21/39) have higher MA (median value = 69.2) 74% (29/31) have higher functional fibrinogen levels (median value = 56 mm) 100% patients had Ly30 = 0% (fibrinolysis shutdown)

*Data are expressed in median and interquartile range. ᶧData are expressed as mean. ᶤData are expressed as mean ± standard deviation (SD). ¶Data are expressed as mean (SD).

R, reaction time, measure of the clotting time from the point of coagulation ignition to the appearance of the clot; K, kinetics, the time from initiation of clot formation to an amplitude of 20 mm; α, alpha angle, denotes velocity of clot formation; MA, maximum amplitude, denotes maximal amplitude of the clot in mm; Ly30,  percentage of clot lysis at 30 minutes after maximum amplitude; TEG, thromboelastography; LCP, laboratory coagulation parameters.

**Supplementary Table 3.** Studies Showing Hypercoagulability Through ROTEM and Laboratory Coagulation Assay

S. No.	Author	ROTEM			Laboratory Coagulation Parameters (LCP)			Incidence of Thrombosis and/or Hypercoagulability Coagulation Parameters (ROTEM/LCP) Associated with Hypercoagulability
EXTEM	INTEM	FIBTEM v	D Dimer (ng mL-1)	Fibrinogen (mg dL-1)	Platelets, (× 109 L-1)
1.	Ibanez et al.^*^ (n = 19)	CT (seconds) 78 (63-91) CFT (seconds) 41 (40-53) MCF (mm) 74 (71-76) LY30% 100 (100-100) LY60 % 99 (97-100)	CT (seconds) 173 (163-201) CFT (seconds) 51 (44-64) MCF (mm) 70 (67-73)	MCF (mm) 30 (24-34) LY30% 100 (100-100) LY 60 % 100 (100-100)	1000 (600-4200)	620 (480-760)	236 (136-364)	7/19 (36.8%) MCF increased in: EXTEM FIBTEM Ly30 and Ly60 increased in: EXTEM FIBTEM D-dimer and fibrinogen increased
2.	Spiezia et alᶤ (n=22)	CT (seconds) 75 ± 16 CFT (seconds) 66 ± 20 MCF (mm) 69 ± 6	CT (seconds) 185 ± 49 CFT (seconds) 57 ± 13 MCF (mm) 68 ± 6	MCF (mm)	5343 ± 2099	517 ± 148	249 ± 119	5/22 (22.7%) CFT decreased in: EXTEM INTEM MCF increased in: EXTEM INTEM FIBTEM.
3.	Almskog et al.^,^ (n = 60)	CT (seconds) 71 (68-75) CFT (seconds) 49 (43-63) MCF (mm) 71 (68-75)	-	MCF (mm) 29 (24-33)	700 (500-1500)	570 (430-690)	221 (181-227)	Incidence of thrombosis not analyzed CT increased in EXTEM MCF increased in EXTEM FIBTEM CFT decreased in EXTEM Platelet count decreased D-dimer and fibrinogen increased
4.	Pavoni et alᶤ (n = 40)	CT (seconds) 78.3 ± 17.2 CFT (seconds) 41.6 ± 11.4 MCF (mm) 76.6 ± 6.4	CT (seconds) 174.6 ± 26.2 CFT (seconds) 38.8 ± 12.1 MCF (mm) 74.9 ± 6.9	MCF (mm) 35.9 ± 5.9	1556 ± 1090	895 ± 110	317.5 ± 168	6/40 (15%) DVT 12/40 (30%) catheter related thrombosis 2/40 (5%) Pulmonary embolism CFT decreased in INTEM: 16/40 (40%) EXTEM: 20/40 (50%) MCF increased in INTEM: 20/40 (50%) EXTEM: 28/40 (70%) FIBTEM: 29/40 (72.5%)
5.	Collett et al^*^ (n = 6)	CFT (seconds) 48.5 (41-60.5) MCF (mm) 74.5 (72.5 -79.5)	CFT (seconds) 39.5 (34.75-51) MCF, mm 75.5 (72.75-77.5)	MCF (mm) 38 (30.5-45.5)	6100 (2585-9660)	750 (720-807)	290.5 (213-338)	2/6 (33%) CFT decreased in: EXTEM (33%) INTEM (83%) MCF increased in: EXTEM (83%) INTEM (83%) FIBTEM (100%)
6.	Nougier et alᶤ (n = 23)	TEM-tPA MCF (mm) 62.3 ± 10 Ly30, % 63 ± 39	-	-	3456 ± 2641	610 ± 190	-	14/23 (60.8%) Ly30% increased (*P* < .029) D-dimer increased

*Data expressed as median and interquartile range. ᶧData are expressed as mean. ᶤData are expressed as mean ± standard deviation (SD). ^¶^Data are expressed as mean (SD).

CT, clotting time; CFT, clot formation time; MCF, maximum clot firmness; Ly30, lysis at 30 minutes; Ly60, lysis at 60 minutes; TEM-tPA, modified ROTEM assay by adding exogenous tPA; EXTEM, extrinsic coagulation pathway; INTEM, intrinsic coagulation pathway; FIBTEM, fibrinogen function.
